# Solution-based synthesis of a CZTS/rGO nanocomposite: structural, electrical and optical studies for solar cell applications

**DOI:** 10.1039/d6ra05065d

**Published:** 2026-09-15

**Authors:** Priyambada Nayak, Pitamber Mahanandia, Jonaki Mukharjee, Subrata Karmakar, Sasanka Sekhar Mishra

**Affiliations:** a Department of Physics, ITER, S‘O’A Deemed to Be University Khandagiri Bhubaneswar Odisha 751030 India priyambada.pce@gmail.com; b Department of Physics & Astronomy, National Institute of Technology Rourkela-769008 Odisha India; c School of Engineering and IT, Arka Jain University Jamshedpur Jharkhand 832108 India; d Department of Physics, Manipal University Jaipur Jaipur Rajasthan 303007 India subrata.karmakar@jaipur.manipal.edu; e Faculty of Pharmaceutical Sciences, S‘O’A Deemed to Be University Khandagiri Bhubaneswar Odisha 751030 India

## Abstract

In the present work, a Cu_2_ZnSnS_4_ (CZTS)/reduced graphene oxide (rGO) nanocomposite was synthesized by a cost-efficient and environmentally friendly solution-based process. The structural analysis based on X-ray diffraction (XRD) indicates that only the CZTS phase is present in the composite system, while Raman spectroscopy and XPS analysis further indicate the presence of both desired phases. The microstructural study provides evidence for the incorporation of rGO on the CZTS material. The optical analysis reveals that the band gap energy (*E*_g_) of the CZTS/rGO nanocomposite system is 1.47 ± 0.02 eV, which is lower than that of the CZTS nanomaterial (*E*_g_ = 1.49 ± 0.05 eV). Additionally, an enhancement of the photoresponse efficiency is observed in the CZTS/rGO nanocomposite system, which was explained on the basis of a reduction in the recombination of charge carriers. Moreover, all the results imply that the synthesized CZTS/rGO nanocomposite system might be an appropriate candidate for solar cell absorber layer applications.

## Abstract 2

Dark and light current plots of the (a) CZTS nanoparticle and (b) CZTS/rGO nanocomposite.

## Graphical abstract

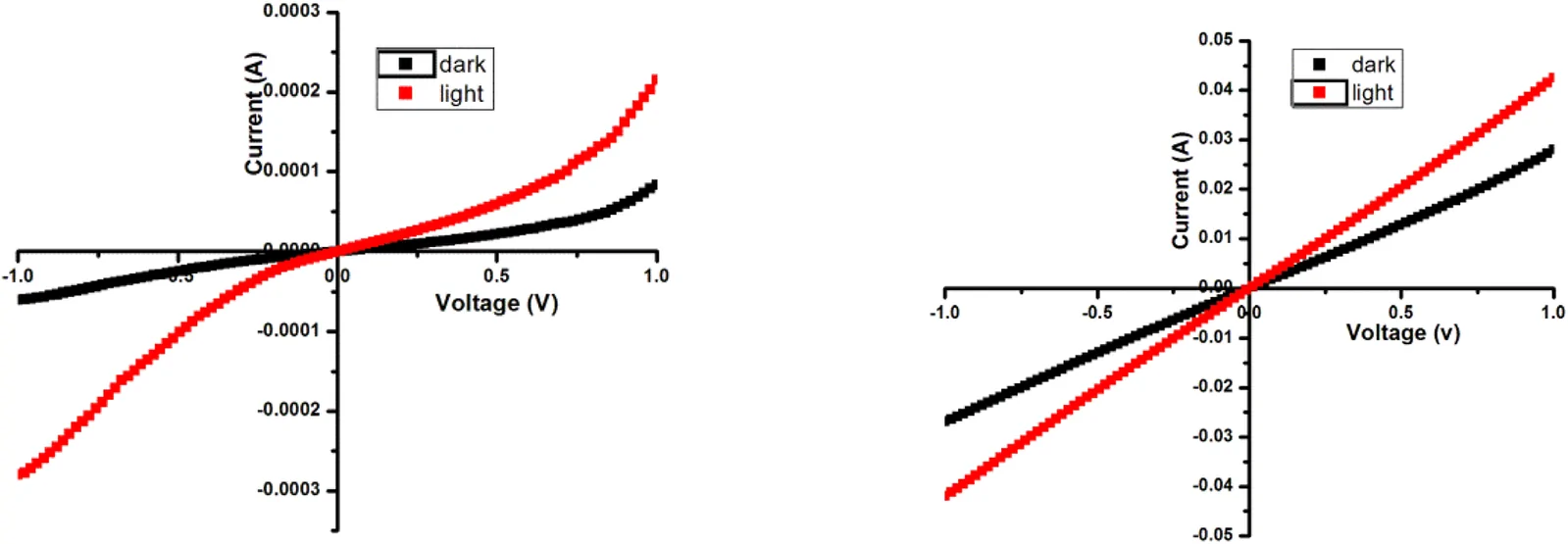

## 1. Introduction

Current research on newly introduced materials for highly efficient and economical solar cells is mainly focused on the constantly growing global energy demands. Hence, considerable research interest has been directed towards the advancement of second-generation solar cells based on semiconducting materials. The development of such materials requires careful consideration of their optical, electronic, and stability-related properties as these characteristics play a crucial role in determining the overall photovoltaic performance. For efficient photovoltaic applications, an ideal solar absorber layer should possess a suitable band gap in the range of approximately 1.0–1.5 eV to ensure effective utilization of the solar spectrum, along with a high optical absorption coefficient, long carrier diffusion length, and excellent chemical and thermal stability. With this strategy, chalcopyrite-type solar cells constructed from absorber materials such as copper indium gallium selenide (CIGS) have gained immense attention owing to their simple fabrication process and scalable production, with a high photoelectron conversion efficiency of 21.7%.^1–3^ However, due to the high cost, limited availability and toxicity of indium (In) and gallium (Ga), research has moved towards alternative earth-abundant compositions that are suitable for large-scale applications in the future.^4–6^ In this context, copper-zinc-tin-sulphur [Cu_2_ZnSnS_4_(CZTS)] has opened up new possibilities involving sulphide materials, which are closely related to structures like CIGS and offer strong absorption coefficients of about ∼10^4^ cm^−1^. These materials have an optical bandgap energy ranging from 1.4 eV to 1.6 eV and can act as p-type conductors suitable for solar cell applications.^7–10^ In addition, CZTS consists of earth-abundant and relatively non-toxic elements, offering advantages over conventional thin-film absorbers based on scarce or toxic elements. Several of these favorable properties have been demonstrated in CZTS-based photovoltaic devices, confirming its potential as a low-cost and sustainable solar absorber. However, the photovoltaic performance of CZTS is still limited by intrinsic defects, secondary phases, carrier recombination, and interface-related losses, which can adversely affect charge transport and carrier collection. Therefore, strategies for improving the optical and electrical properties of CZTS are essential for enhancing its photovoltaic performance.

The Shockley–Queisser limit photon balance calculations predict that the theoretical power conversion efficiency (PCE) of a CZTS-based solar cell is approximately 32.2%.^11^ Based on this concept, different research groups have attempted to increase the PCE of CZTS from 0.66% to 11.1% through optimization of the fabrication process. In short, Katagiri *et al.* fabricated the CZTS material by using a co-sputtering technique and reported a promising conversion efficiency of 6.77%.^12^ Further study revealed a photoelectric conversion efficiency enhancement to 7.37% by selenization of the CZTS thin film.^13^ Y. Feng also achieved a power conversion efficiency (PCE) of 8.4% for CZTS solar films that were grown by an evaporation process.^14^ Researchers from IBM achieved a record efficiency of 9.6% for a copper zinc tin sulfide (CZTS) solar cell that was manufactured by using a solution-based spin-coating technique combined with heat treatment.^15^ Previously reported results suggested that Cu–Zn antisite defects, such as [Cu_Zn_^−^ + Zn_Cu^+^_] are the major factor limiting the power conversion efficiency (PCE) of kesterite CZTS-structured solar cells.^16^ Meanwhile, both the crystal structure and elemental stoichiometric ratio are key factors that significantly influence the electrical as well as optical properties of CZTS materials. To overcome these issues, different strategies are actively being studied by adopting two main approaches. First, the most commonly proposed method aims to reduce Sn during the annealing process.^17^ Secondly, many researchers attempt to prepare CZTS-based thin films.^18^ However, the main drawback of the CZTS thin-film approach is that when incident photons of higher energy are absorbed within a short distance from the surface, high recombination loss occurs. Furthermore, the built-in electric field can be enhanced by improving the absorption of incident light. In bulk materials, light absorption exhibits exponential behavior, and a thick absorber layer is required to generate more carrier generators. However, when the absorber thickness is more than the carrier diffusion length, charge recombination becomes dominant. Therefore, improvements in synthesis methods and nanofabrication techniques, together with controlled nanoscale engineering of materials, are essential for enhancing the power conversion efficiency (PCE) of solar energy conversion devices. However, the low carrier mobility of nanomaterials continues to limit their optical performance in practical applications. Therefore, an alternative approach is to develop composite systems by combining CZTS nanomaterials with a material having high electron mobility.

In this regard, ultrafast charge-carrier transport materials with high mobility are used, such as graphene, which is a carbon allotrope generally used to improve the PCE due to its remarkable properties of high carrier mobility (value), high specific surface area, and excellent optical transparency and conductivity. Enhanced light absorption and superior charge-transfer characteristics with high quantum efficiency were observed in graphene-based hybrid systems, as reported by Gerasimos Konstantatos *et al.* (∼25%).^19^ Additionally, a graphene derivative identified as reduced graphene oxide (rGO) has received attention in photodetection applications because of its fantastic physical properties.^20^ For example, an improved power conversion efficiency (PCE) of 7.81% was described by Bai *et al.* for hybrid CZTS/rGO dye-sensitized solar cells (DSSCs).^21^ But, unfortunately, the potential instability, temperature sensitivity and leakage caused by the liquid electrolyte used in DSCCs limit their applicability. Similarly, Thangaraju *et al.* described an enhancement in the optical properties of the CZTS/rGO system.^22^ Therefore, from the real-world applications point of view, very few research papers are available that feature a detailed investigation of electrical and optical properties of CZTS/rGO nanocomposite systems.^23,24^ It is expected that the synergistic effects of the CZTS/rGO nanocomposite materials may overcome the drawbacks to some extent. In the hybrid system, the nanomaterials serve as light-absorbing components that produce electron–hole pairs under light irradiation. The generated carriers are then rapidly transferred to the reduced graphene oxide sheets, resulting in efficient electron–hole separation, enhanced photocurrent generation, and longer carrier lifetimes. Therefore, the effective transfer of photogenerated carriers to the rGO surface contributes to high optical gain and excellent photoresponse behavior. Motivated by these advantages, we have synthesized a copper zinc tin sulfide/rGO nanocomposite using a low-cost, effective, scalable, and environmentally friendly solution-based synthesis method. The structural, microstructural, electrical and optical properties are analyzed in detail, which would be helpful for constructing solar cell absorbers.

## 2. Materials and methods

The solution-based synthesis route offers numerous advantages, including low processing cost, simple handling and ease of preparation. Therefore, to prepare the nanocomposite material, copper(ii) chloride dihydrate (99%), zinc chloride (98%), stannous chloride dihydrate (97%) and thiourea (99.6%) were procured. The copper zinc tin sulfide (CZTS) precursor solution was prepared using a solution-based method by dissolving CuCl_2_·2H_2_O (0.01 M), ZnCl_2_ (0.0078 M), SnCl_2_·2H_2_O (0.0056 M), and CH_4_N_2_S (0.0444 M) in ethanol and distilled water. The corresponding molar ratio of Cu : Zn : Sn : S precursors was maintained at 0.01 : 0.0078 : 0.0056 : 0.0444, respectively, to obtain the desired CZTS nanoparticles. The prepared solution was then stirred continuously for 4 h to obtain a uniform and stable suspension. After that, the precursor was annealed at 150 °C to 500 °C in a programmable furnace to achieve pure-phase CZTS nanoparticles. Graphene oxide (GO) was synthesized by a modified Hummers process and consequently reduced to achieve reduced graphene oxide (rGO). In short, the prepared GO was then ultrasonicated to exfoliate the GO sheets, followed by chemical reduction using hydrazine hydrate. The resulting rGO was then neutralized, washed thoroughly with deionized water several times, and finally dried in vacuum at 100 °C to achieve the required rGO flakes. The synthesized rGO and CZTS nanoparticles were characterized separately. To prepare the nanocomposite, rGO and CZTS were separately dispersed in *N*,*N*-dimethylformamide (DMF) and separated using ultrasonication and magnetic stirring. The two stable dispersions were mixed and again sonicated for 2 hours and centrifuged with an equal volume of ethanol. The mixed dispersion was then dried at 80 °C under vacuum to obtain the CZTS/rGO nanocomposite.

The crystallographic structure and phase identification of the prepared CZTS/rGO nanocomposites were studied using a Rigaku Ultima IV (Japan) X-ray diffractometer (XRD) with CuK_α_ radiation (*λ* = 1.541 Å) over a 2*θ* range of 20°–80° at a step size of 0.02°. The Raman scattering measurements were recorded in backscattering geometry using a Jobin–Yvon T64000 triple-monochromator equipped with a large coupled detector, using a 532 nm excitation wavelength over the spectral range of 100–1000 cm^−1^. The valence states of the constituent elements in the prepared samples were analyzed using a Physical Electronics X-ray photoelectron spectrometer (PHI 5082). The microstructural features and surface characteristics of the nanocomposite material were analyzed with a scanning electron microscope (JEOL JSM-6084 LV) operated at an accelerating voltage of 10 kV. TEM analysis was conducted at an accelerating voltage of 200 kV (TEM, Tecnai G220); a few milligrams of the sample were dispersed in isopropyl alcohol (0.2 g mL^−1^), followed by ultrasonication for 20 min, and a few drops of the resulting suspension were deposited onto a holey carbon grid. The crystal structure was further analyzed based on the selected-area electron diffraction (SAED) patterns. The band gap energy was obtained using a UV–vis spectrophotometer (Shimadzu UV-3600). The photocurrent–voltage (*I*/*V*) characteristics of the prepared sample were recorded with a KEITHLEY 2400 Source measurement unit at room temperature, controlled by a computer under 100 mW cm^−2^ illumination with a standard halogen lamp.

## 3. Results and discussion

### 3.1. Structural characterization (XRD)

To gain details of the electrical and optical characteristics, it was important to first establish the phase purity and crystallinity of the prepared CZTS material. Fig. 1a displays the XRD (X-ray diffraction) patterns of the prepared CZTS nanoparticles annealed at different temperatures (150 °C–500 °C). The results clearly confirmed that a single-phase kesterite structure was formed, with no evidence of any secondary impurity phases, such as binary or ternary sulfide phases. The diffraction peaks are marked with asterisks (*) corresponding to the (112), (200), (220), (312), (324), (008) and (332) planes at 2θ values of 28.5°, 33.0°, 47.3°, 56.1°, 59.0°, 69.2° and 76.3°, respectively. These peaks were indexed to the *I*4̄2*m* space group in accordance with JCPDS card No. 26-0575 and are in good agreement with the previous reports.^25,26^

**Fig. 1:**
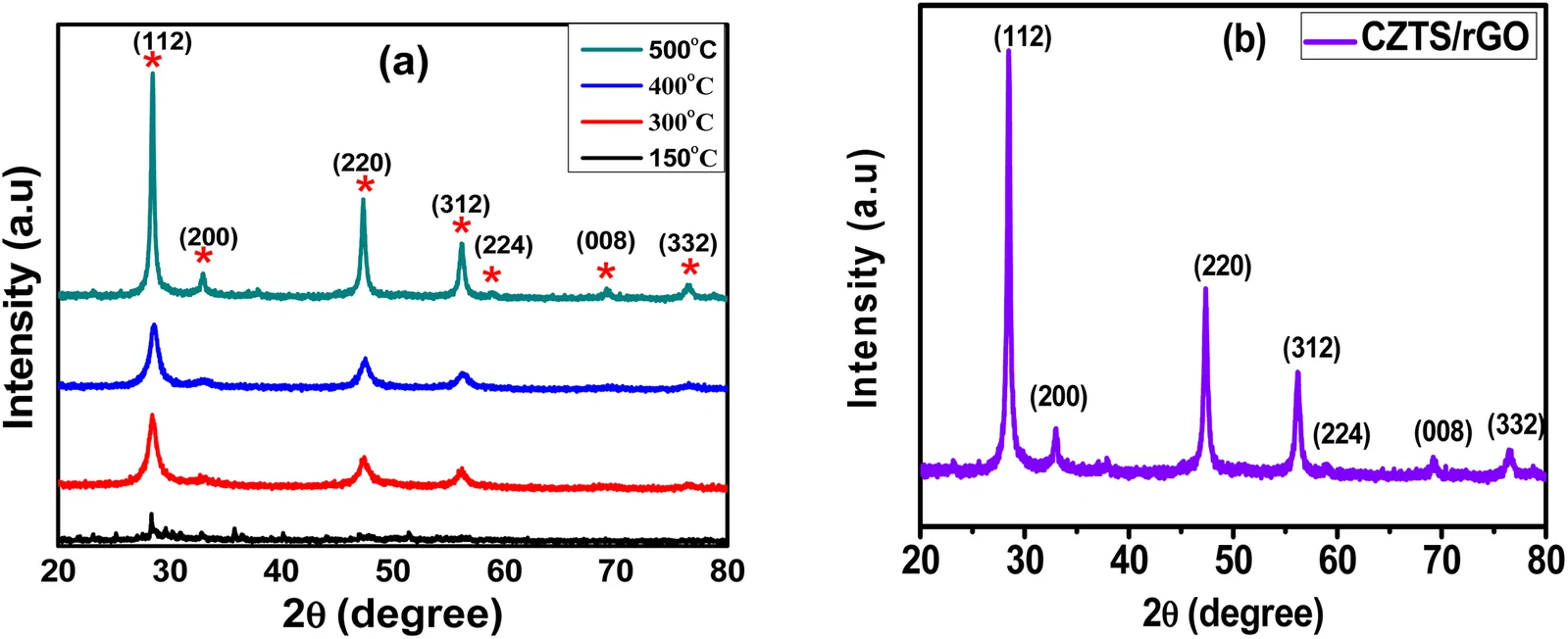
(a) XRD patterns of the CZTS nanoparticles annealed at different temperatures. (b) XRD pattern of the CZTS/rGO nanocomposite.

However, from Fig. 1a, it is observed that with an increase in annealing temperature, the peak intensity gradually increases, whereas the peak full-width at half-maximum (FWHM) decreases. The diffraction peaks of the sample annealed at 500 °C are more intense and sharper than those of other samples. This enhancement indicates improved crystallinity, increased long-range atomic ordering, and a reduction in structural defects. The decrease in FWHM values with the increase in annealing temperature again signifies grain growth, a reduction in microstrain, and relaxation of internal stresses. Therefore, the calculated parameters, such as crystallite size, lattice strain and dislocation density, highlight the strong influence of thermal energy on the crystallization behavior and are summarized in Table 1. The crystallite size and lattice strain were estimated using the Debye–Scherrer equation provided below:^27^1
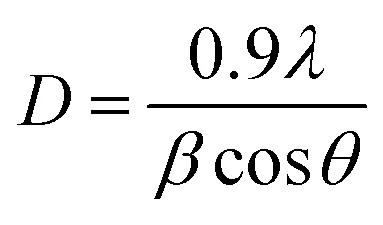
2
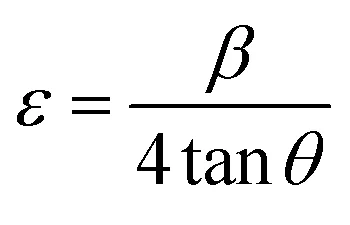
where ‘*λ*’ refers to the wavelength of the X-ray for CuKα radiation (1.5406 Å), ‘*β*’ refers to the full-width at half-maximum (FWHM) and *θ* denotes the angle of diffraction.^28^ The estimated values of the average crystallite sizes for the CZTS nanoparticles annealed at 150 °C, 300 °C, 400 °C, and 500 °C are 19.2 nm, 15.12 nm, 10.6 nm and 8.15 nm, respectively. These results indicate that the annealing temperature plays a vital role in the formation of the CZTS nanoparticles. Again, the microstrain gradually decreases, which indicates effective thermal relaxation. Therefore, an increase in annealing temperature enables trapped defects, dangling bonds, and microstresses to reorganize or annihilate, resulting in a more uniform crystal lattice. Furthermore, the dislocation density is defined as the total length of dislocation lines per volume, and it is a key indicator of the defect concentration within a crystal. These dislocations, which arise from lattice irregularities or mismatches, typically originate from strain or imperfections present during the material synthesis. The dislocation density (*δ*) was determined using the following equation:

3 *δ* = 1/*D*^2^

**Table 1:** Calculated structural parameters of the CZTS nanoparticles

Structural parameter	150 °C	300 °C	400 °C	500 °C
Average crystallite size (nm)	19.2	15.12	10.61	8.15
Lattice strain (*ε*)	0.0069	0.0067	0.0064	0.0061
Dislocation density (*δ*) × 10^−3^ (nm^−2^)	2.77	4.47	8.88	15.05

It follows the inverse trend of the crystallite size, *i.e.*, the minimum *δ* is observed at 100 °C (2.77 × 10^−3^ nm^−2^), where the crystallite size is the largest, and the maximum *δ* is observed at 500 °C (15.05 × 10^−3^ nm^−2^), reflecting a reduced crystallite size. This confirms that annealing up to 100 °C–500 °C improves the crystal quality. Meanwhile, at 500 °C, recrystallization and renucleation processes cause an increase in crystallinity, with the development of small nuclei that grow on their own and then merge with the more regular ones. Many crystallites are created during the annealing process; this increases the absorption surface area and leads to a reduction in the optical band gap by causing more light to be absorbed than transmitted. Thus, 500 °C was selected as the optimum annealing temperature for further studies. Again, in Fig. 1b, the nature of these peaks does not exhibit any significant change with the addition of rGO into the host CZTS material. This indicates that the structure remains unaffected during the formation of the CZTS/rGO nanocomposite. Therefore, it can be concluded that the prepared material may show composite behavior, as will be further studied using Raman analysis in the next section.

### 3.2. Raman analysis

It is challenging to identify the secondary phases of Cu_2_S, SnS and ZnS in the CZTS system from the XRD analysis due to its limited detection sensitivity. Hence, Raman spectroscopy was performed to determine whether secondary phases were present in the CZTS material. Fig. 2a displays the room-temperature Raman spectra of the CZTS nanoparticles annealed at different temperatures. The obtained spectra exhibit a prominent peak at 332 cm^−1^, assigned to the kesterite phase of the CZTS sample, which is consistent with previous results.^28^ The intense peak observed at 332 cm^−1^ is attributed to the A1 vibrational mode, which stems from the vibration of sulphur atoms while the other atoms in the CZTS lattice remain fixed.^29^ The weaker peak appearing at 252 cm^−1^ corresponds to the B (TO/LO) and E (TO/LO) vibrational modes, which are generally identified through polarization-dependent Raman measurements based on selection rules.^30^ It can also be noted that, after annealing at 500 °C (see Fig. 2a), the main peaks at 332 cm^−1^ become sharper due to improved crystallinity. The absence of characteristic peaks related to cubic CTS (295–303 and 355 cm^−1^), tetragonal CTS (297, 337 and 352 cm^−1^), orthorhombic CTS (318 cm^−1^), cubic ZnS (267, 303 and 356 cm^−1^) and Cu_2_−*x*S (264 and 475 cm^−1^) further confirms the formation of phase-pure kesterite CZTS.^26^ Additionally, no noticeable peaks corresponding to SnS_2_ (215 and 315 cm^−1^) were observed.^31^Fig. 2b shows that the CZTS/rGO nanocomposite exhibits prominent D and G bands at 1346 cm^−1^ and 1576 cm^−1^, respectively, indicating the successful incorporation of rGO into the composite. The D band is associated with the disordered structure of sp^2^-bonded carbon, while the G band represents the E_2g_ vibrational mode of graphitic carbon.

**Fig. 2:**
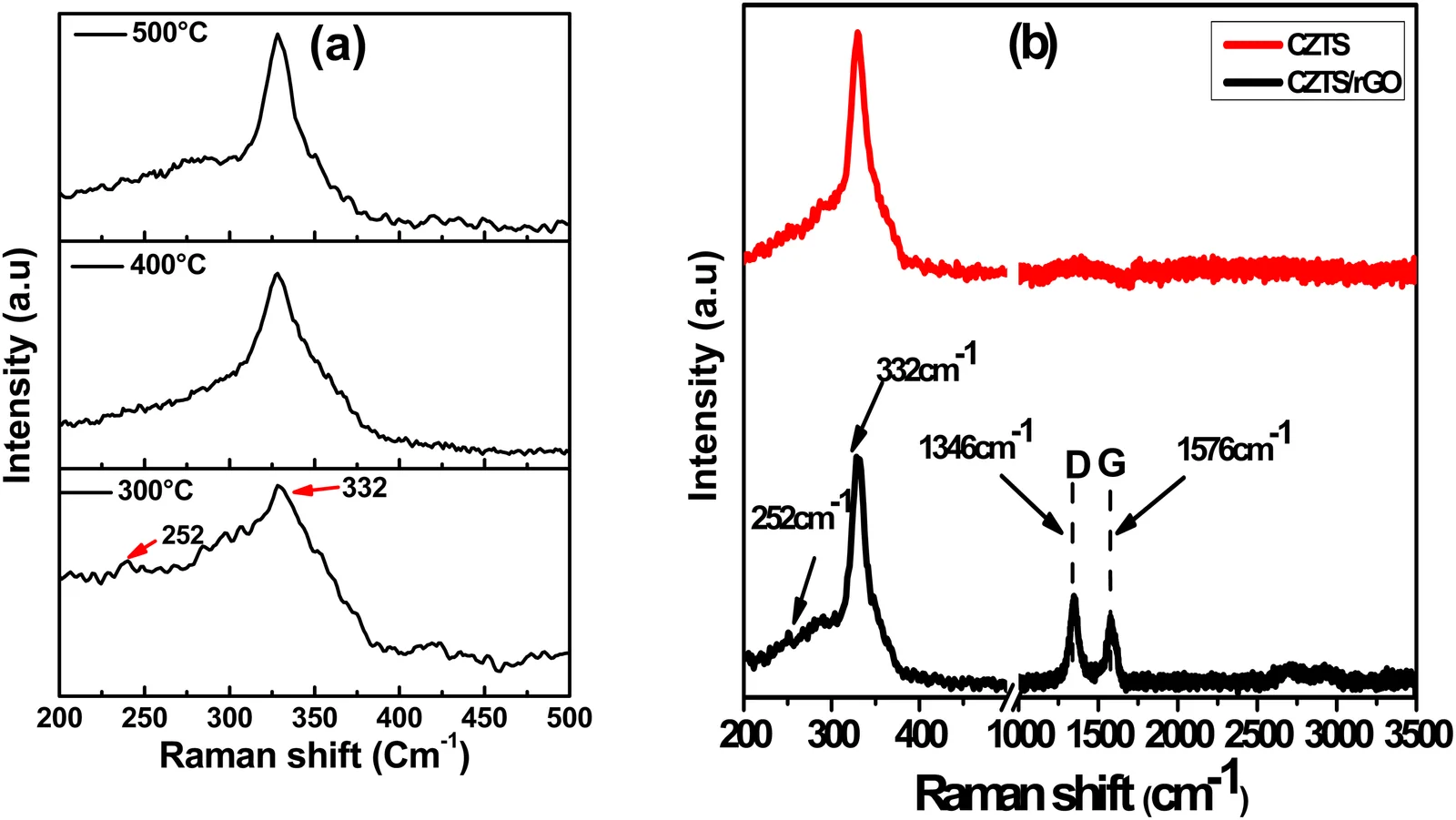
Raman spectra of the (a) CZTS nanoparticles with different annealing temperatures and (b) CZTS nanocomposite annealed at 500 °C and CZTS/rGO nanocomposite.

### 3.3. XPS analysis

The valence states of the constituent elements present in the synthesized CZTS annealed at 500 °C and the CZTS/rGO nanocomposite were evaluated using XPS analysis, as presented in Fig. 3. The XPS wide scans for the CZTS and CZTS/rGO nanocomposites show peaks of S 2p, Cu 2p, Zn 2p and Sn 3d, along with a C 1s peak at 284 eV (Fig. 3a and b). Moreover, in the composite samples, the C 1s peak becomes prominent with an increase in intensity due to the presence of rGO. The S 2p core-level spectra in Fig. 4a and b exhibit two binding energy peaks located at 160.8 eV and 162 eV, corresponding to S (2p_3/2_) and (2p_1/2_), respectively. The energy separation of 1.2 eV confirms the presence of sulphur in the sulphide phase with a −2 oxidation state.^32,33^ The Cu 2p core-level spectra (Fig. 4c and d) display two characteristic peaks at 931.9 eV (2p_3/2_) and 951.6 eV (2p_1/2_), indicating the existence of Cu in the +1 oxidation state, which arises from the reduction of Cu^2+^ in the present material.^34^

**Fig. 3:**
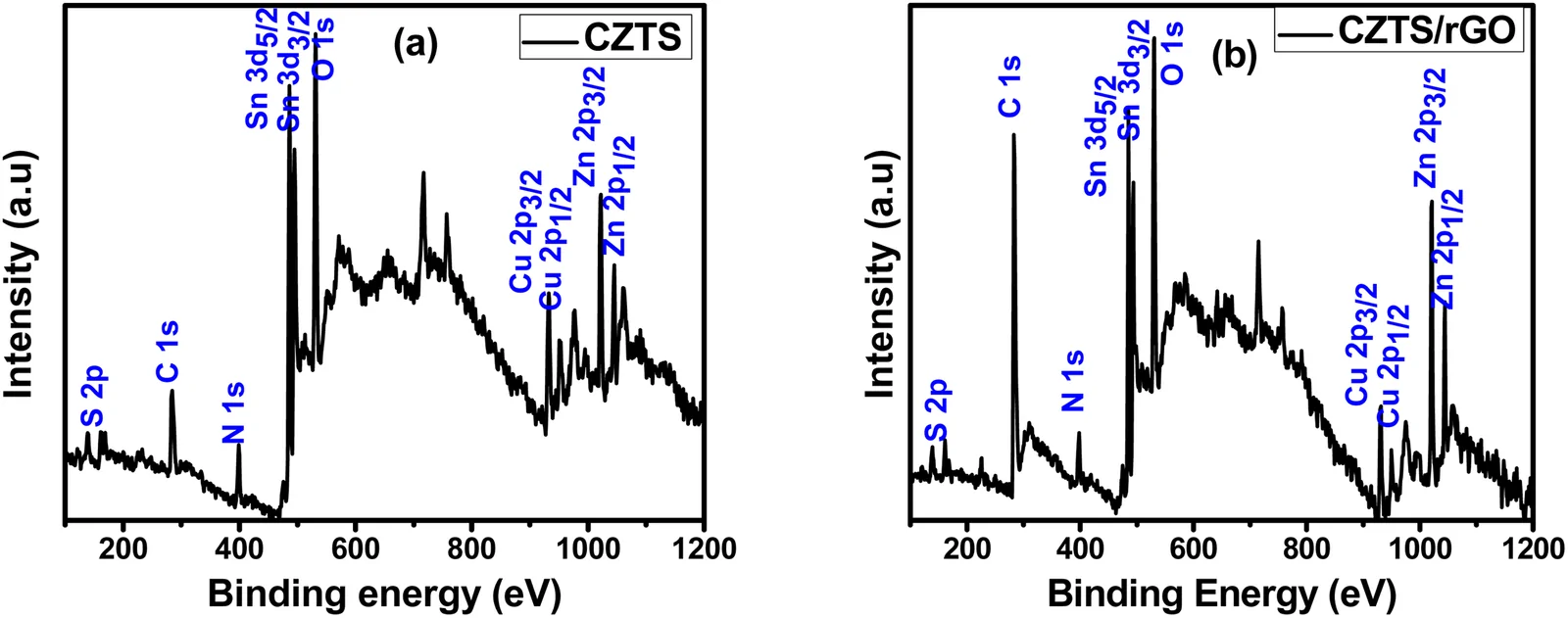
XPS wide scan spectra of the (a) CZTS nanoparticles annealed at 500 °C and (b) CZTS/rGO nanocomposite.

**Fig. 4:**
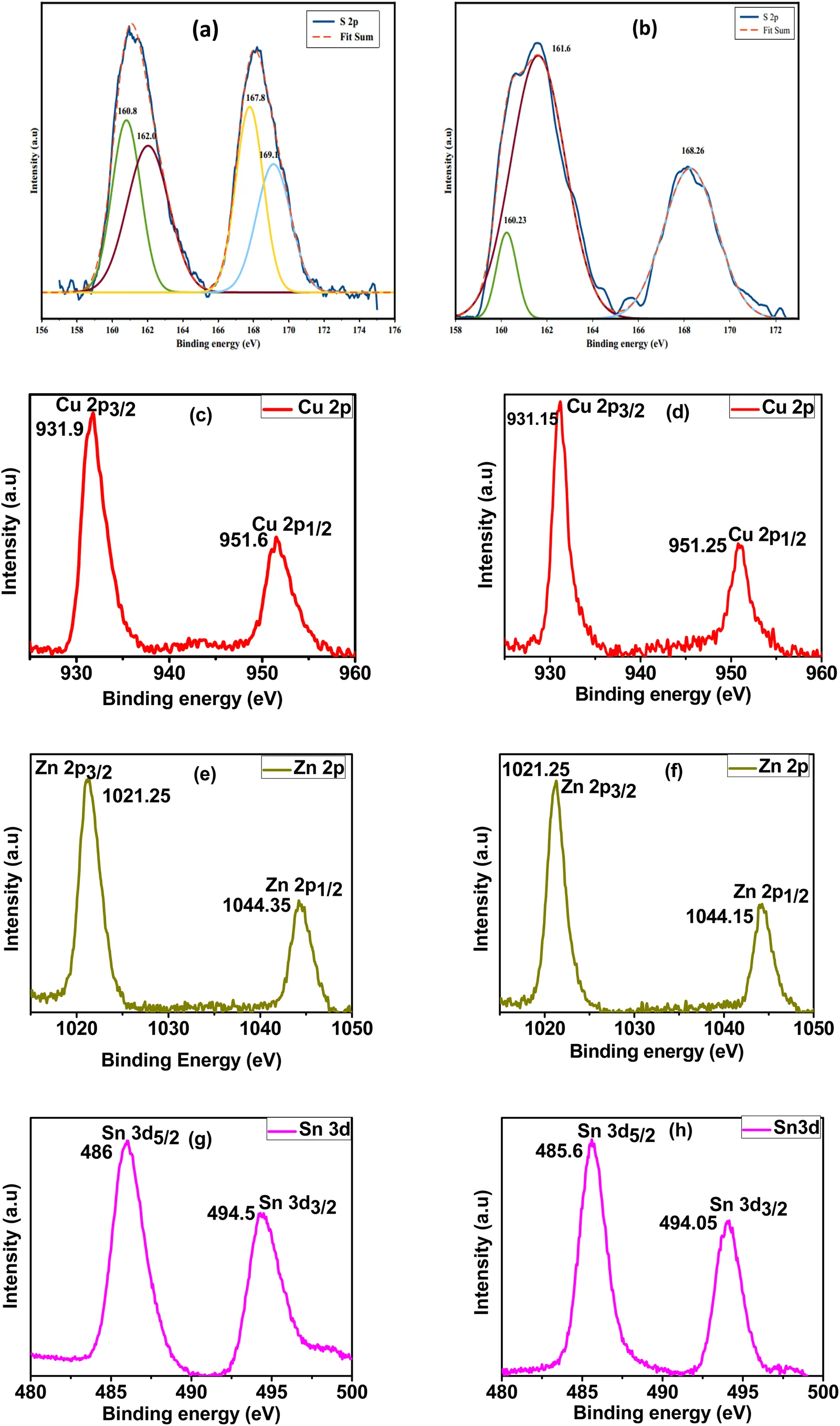
XPS core-level spectra of (a) S 2p, (c) Cu 2p, (e) Zn 2p and (g) Sn 3d of the CZTS nanoparticles annealed at 500 °C. XPS core-level spectra of (b) S 2p, (d) Cu 2p, (f) Zn 2p and (h) Sn 3d of the CZTS/rGO nanocomposite.

Similarly, the Zn 2p spectra shown in Fig. 4e and f and Sn 3d spectra in Fig. 4g and h also exhibit characteristic peak splitting. The peaks located at 1021.25 eV and 1044.35 eV correspond to Zn (Zn 2p_3/2_) and (Zn 2p_1/2_), respectively, while the peaks at 486 eV and 494.5 eV are assigned to Sn 3d_5/2_ and Sn 3d_3/2_, respectively. These results confirm the presence of Zn in +2 and Sn in +4 oxidation states, with energy separation values of 23.1 eV and 8.5 eV, respectively.^35^

### 3.4. Microstructural analysis

Fig. 5a shows a SEM image of the CZTS nanoparticles annealed at 500 °C that indicates the formation of uniformly distributed spherical particles that agglomerate and form clusters because of their small size. The TEM and HRTEM images of the CZTS nanoparticles are shown in Fig. 5b and c, which indicate that the particles are nanometer-sized. The value matches somewhat well with the JCPDS 26-0575 file for the *d*-spacing of the preferred orientation (112) plane of CZTS crystals discussed above. The HRTEM image also shows typical fringes, illustrating that the kesterite CZTS is crystalline in nature. The SAED (selected area electron diffraction) pattern shown in Fig. 5d reveals that the CZTS nanoparticle exhibits many diffraction rings corresponding mainly to the (112) and (220) planes. Fig. 5e displays the SEM image of the CZTS/rGO nanocomposite, which shows CZTS particles uniformly covering the rGO sheets. Similarly, Fig. 5f and g show TEM and HRTEM images of the CZTS/rGO nanocomposite, which confirm the uniform arrangement of the CZTS particles on the rGO sheets. The SAED pattern shown in Fig. 5h also displays good crystallinity, with fine concentric rings corresponding to the (112), (220) and (312) planes of the CZTS nanoparticle and the (002) plane of the rGO sheet.

**Fig. 5:**
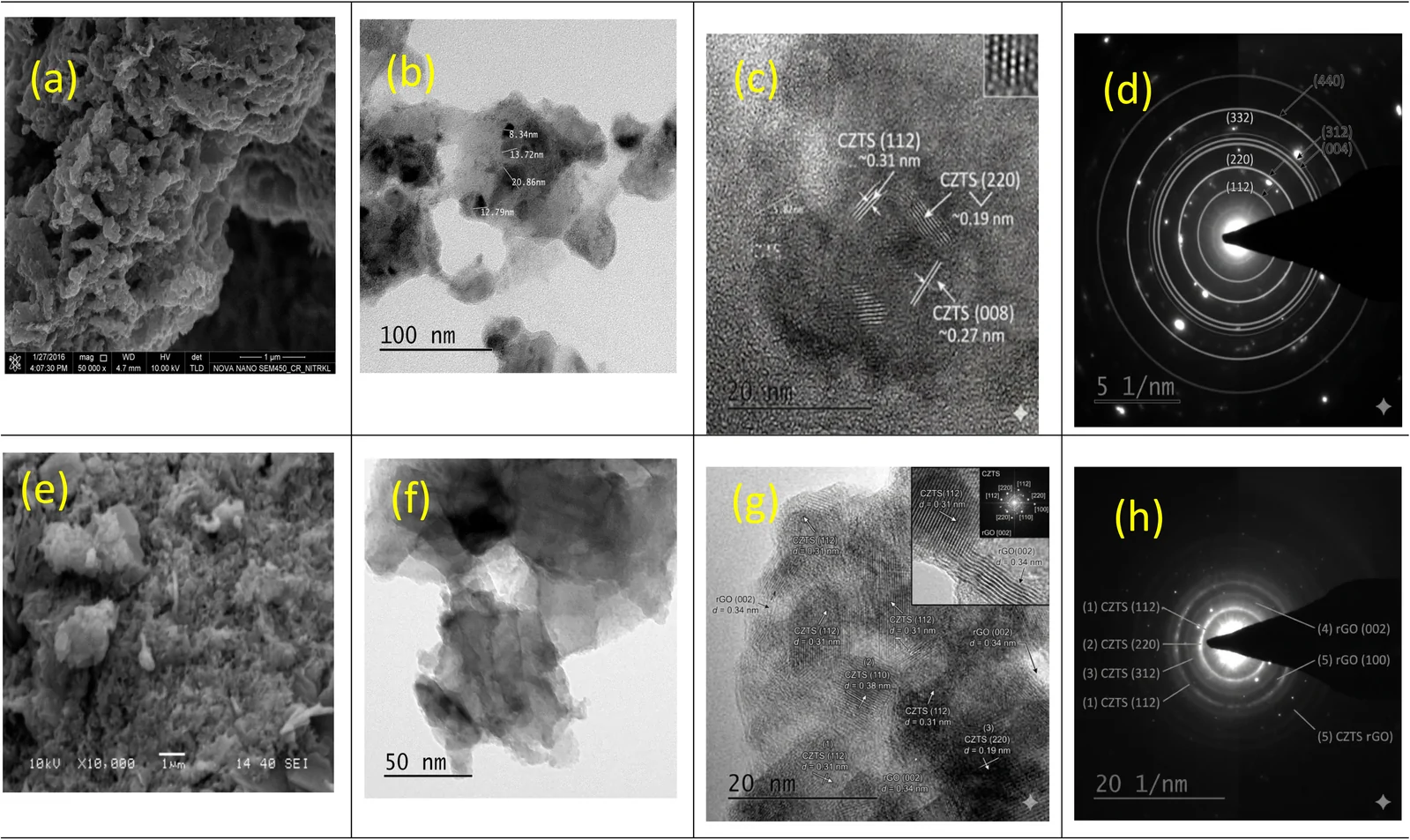
(a) SEM micrograph, (b) TEM image, (c) HRTEM image and (d) SAED pattern of the CZTS nanoparticles annealed at 500 °C. (e) SEM micrograph, (f) TEM image, (g) HRTEM image and (j) SAED pattern of the CZTS/rGO nanocomposite.

### 3.5. Optical absorption

For solar cell applications, the prepared CZTS/rGO nanocomposite was characterized by an optical study. To this end, the nanocomposites were examined by using diffuse reflectance spectroscopy. The optical band gap energy (*E*_g_) value was determined from the fundamental absorption associated with the excitation electron switch from the valence band (VB) to the conduction band (CB). Therefore, the reflectance data was converted into absorbance by the Kubelka–Munk function as follows:4
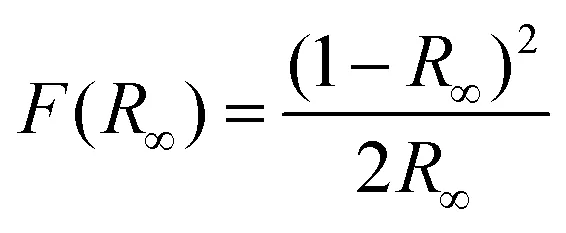
where *R*_∞_ signifies the diffuse reflectance and *F*(*R*_∞_) is the Kubelka–Munk function corresponding to the absorbance (*α*).^36,37^Fig. 6a depicts the absorption band spectra of the CZTS and CZTS/RGO nanocomposite system. Both samples exhibit broad absorption behavior in the visible region, with the absorption tails extending toward longer wavelengths. It is observed that there is a red shift in the absorption spectra with rGO incorporation in the CZTS material. Furthermore, the band gap energy (*E*_g_) for the composite systems can be estimated from the Tauc equation. According to that, for materials with a direct band gap, the absorption coefficient is calculated as follows:^38^

5 α*hυ* = Cont. (*hυ* − *E*_g_)^*n*^

where *hυ* refers to the energy of a photon, *α* denotes the coefficient near the absorption edge and *n* is an exponent that describes the optical absorption progression (*n* = 2 for indirect band gap and *n* = 1/2 for direct band gap).^3^ As CZTS performs as a direct band gap semiconductor, the band gap was approximated by plotting (α*hυ*)^2^*vs.* the energy (*hυ*), as shown in Fig. 6b and c. Extrapolation of the linear portion of the (α*hυ*)^2^*vs.* (*hυ*) plots gives the value of *E*_g_. The calculated bandgap energy *E*_g_ for the CZTS/rGO nanocomposite is 1.47 ± 0.02 eV, which is slightly lower than that of the bare CZTS material (*E*_g_ = 1.49 ± 0.05 eV). The marginal reduction in the band gap (*E*_g_) of the CZTS/rGO nanocomposite may be associated with strong interfacial coupling and electronic interaction between the CZTS phase and the rGO matrix. Such interactions may generate additional localized defect states and promote interfacial charge–transfer processes, thereby modifying the electronic density of states near the band edges. As a consequence, the absorption threshold undergoes a slight redshift toward longer wavelengths, resulting in an extended optical absorption range and improved light-harvesting capability. Hence, the movement of electrons from the valence band to the conduction band becomes easier. Again, the constant is an indicator of the degree of structural disorder for semiconducting materials. The value of *C* was evaluated from the slope of the fitting. Low values of *C* indicate more structural disorder, whereas higher values of *C* indicate a reduction in structural disorder.^39^ In this case, the higher *C* value shows less structural distortion.

**Fig. 6:**
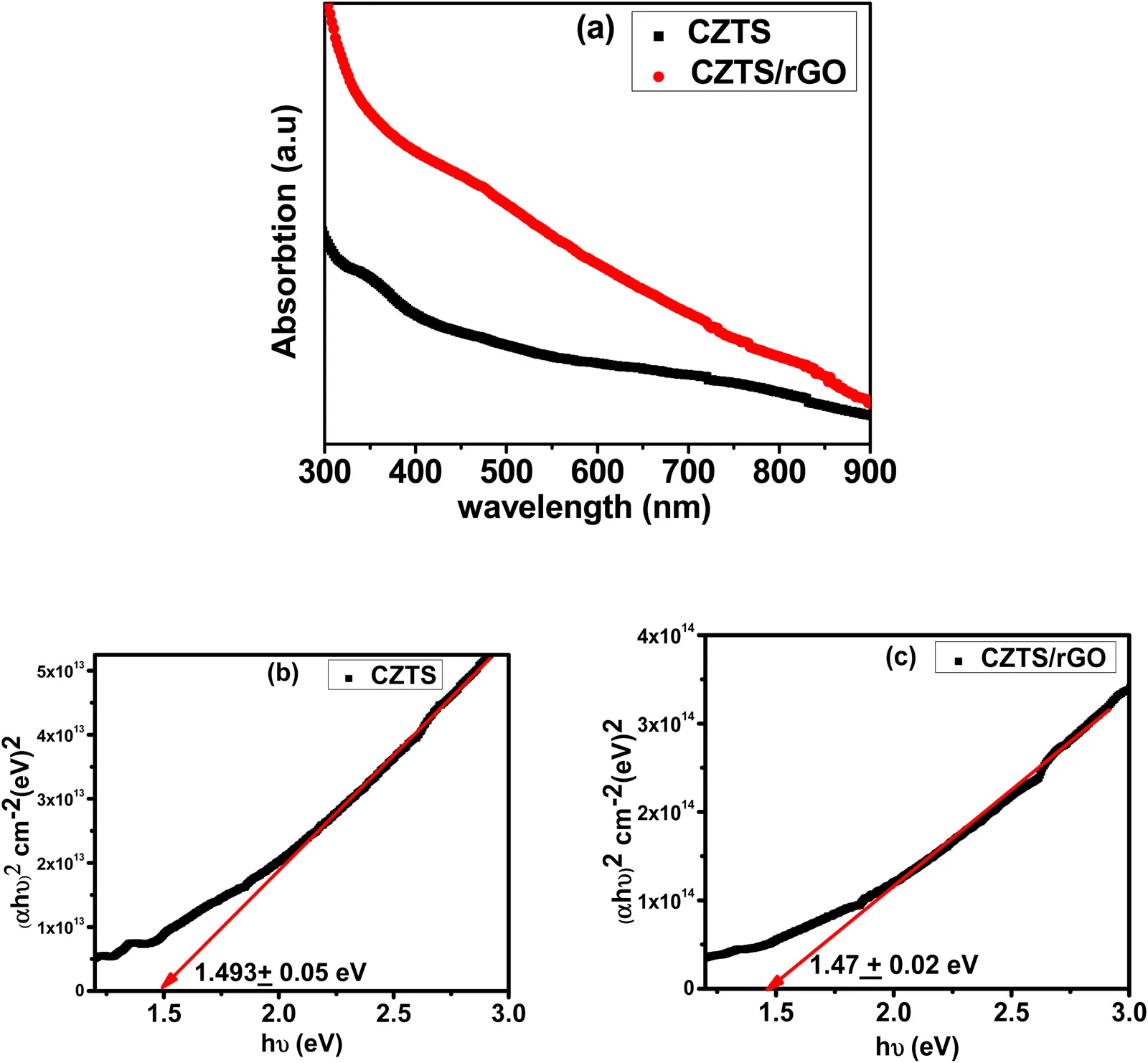
(a) Room-temperature UV-visible absorption spectra of the CZTS nanoparticles and CZTS/rGO nanocomposite. Tauc plots of the (b) CZTS and (c) CZTS/rGO nanocomposites.

### 3.6 . Photoelectric response

For photovoltaic applications, the photoresponse behavior of the CZTS and CZTS/rGO composites was investigated. Fig. 7 shows the *I*–*V* characteristic curves of both samples recorded under dark and illuminated conditions using a solar simulator. Both CZTS and CZTS/rGO nanocomposites exhibit nearly linear and symmetric current–voltage curves about the origin. An increase in applied voltage resulted in a corresponding increase in current, indicating the ohmic nature of the electrical contacts. Furthermore, both samples showed enhanced current under illumination, similar to the behavior reported for other semiconductor nanocrystals.^28,40^ The photocurrent (*I*_ph_) can be determined from the difference between the illuminated current (*I*_L_) and dark current (*I*_D_) as given by:

6 *I*_ph_ = *I*_light_ − *I*_dark_

**Fig. 7:**
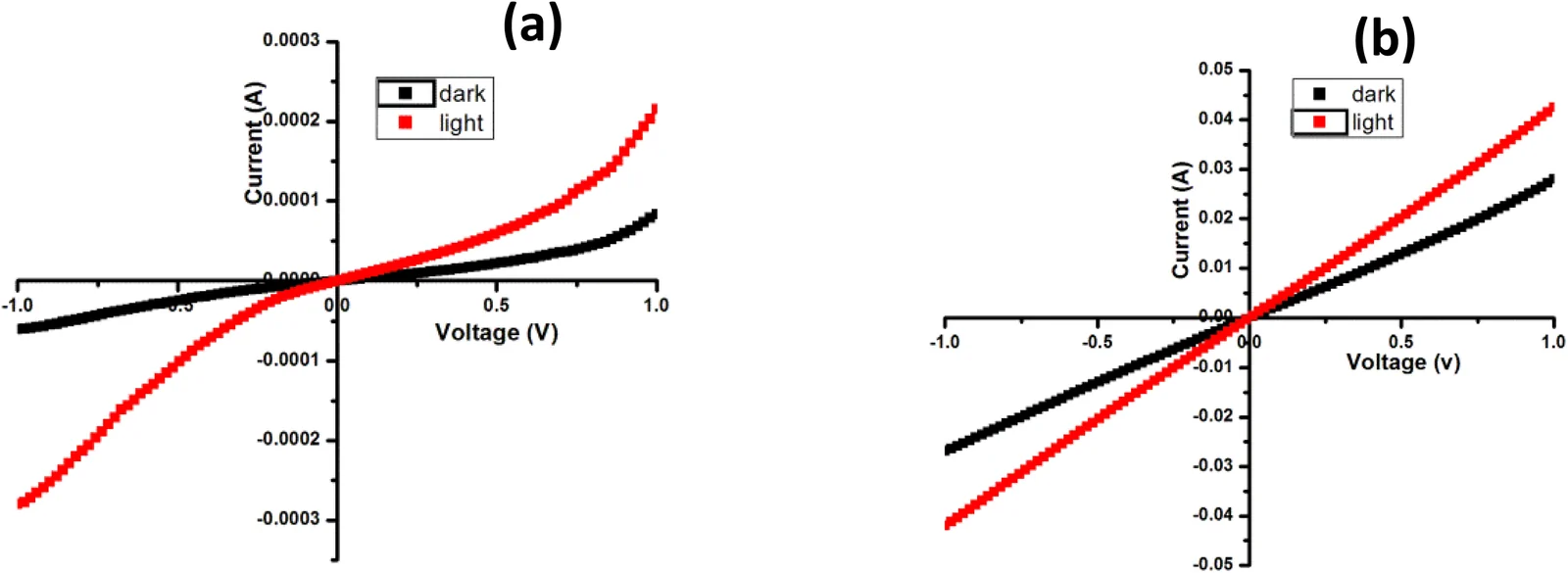
Dark and light current plots of the (a) CZTS nanoparticles and (b) CZTS/rGO nanocomposite.

At an applied voltage of 1 V, the CZTS sample exhibits a dark current of approximately 8 × 10^−5^ A and an illuminated current of approximately 2.1 × 10^−4^ A, resulting in a photocurrent of about 1.3 × 10^−4^ A. In comparison, the CZTS/rGO nanocomposite shows a significantly enhanced current, increasing from approximately 0.027 A in the dark to 0.042 A under illumination, corresponding to a photocurrent of approximately 0.015 A. The photoresponse can also be evaluated using the light-to-dark current ratio, expressed as follows:^41^7
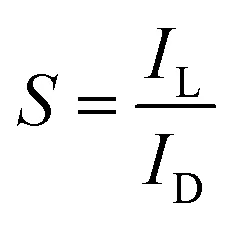


The calculated light-to-dark current ratios are approximately 2.6 and 1.56 for CZTS and CZTS/rGO, respectively. Furthermore, the percentage enhancement in current under illumination can be estimated using the following relation:^42^8
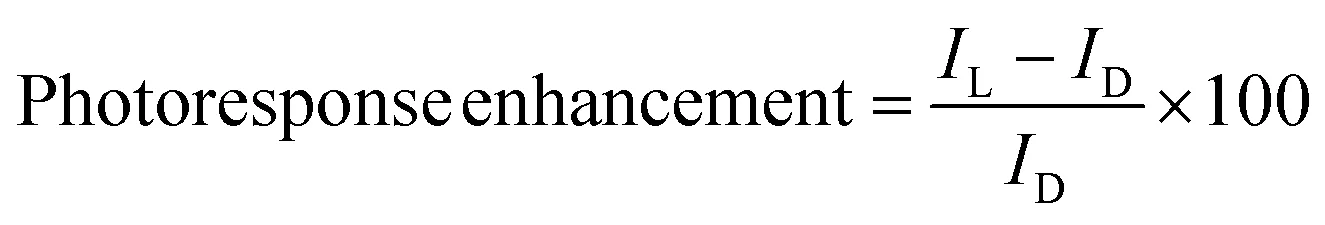


Accordingly, the estimated photoresponse enhancement is approximately 162% for CZTS and 56% for CZTS/rGO at 1 V. Despite the lower percentage enhancement, the CZTS/rGO nanocomposite exhibits a substantially higher absolute photocurrent than pristine CZTS, indicating improved charge transport following rGO incorporation. Furthermore, the enhancement in photocurrent under illumination can be attributed to the excitation of electrons from the valence band (VB) to the conduction band (CB), resulting in the generation of electron–hole pairs and an increased concentration of photogenerated charge carriers. Consequently, the photoconductivity of both CZTS and CZTS/rGO is enhanced under illumination. In the CZTS/rGO nanocomposite, the highly conductive rGO network provides additional pathways for the efficient transport and extraction of photogenerated charge carriers. Upon illumination, photogenerated electrons from CZTS can be efficiently transferred to the rGO sheets, where they can be transported rapidly through the conductive carbon network. This efficient interfacial charge transfer promotes electron–hole separation and suppresses their recombination, thereby enhancing the photocurrent response. The intimate contact between CZTS and rGO further facilitates interfacial charge separation and improves carrier extraction. Moreover, the slight reduction in the optical band gap from 1.49 ± 0.05 eV for CZTS to 1.47 ± 0.02 eV for CZTS/rGO may contribute to slightly enhanced light absorption and photocarrier generation. Furthermore, the incorporation of rGO results in an approximately 10^2^-fold increase in photocurrent compared with bare CZTS under illumination, highlighting the significant improvement in charge transport and collection. Therefore, the synergistic effects of enhanced photocarrier generation, efficient interfacial charge separation, reduced electron–hole recombination, and improved carrier transport collectively contribute to the superior photoresponse of the CZTS/rGO nanocomposite.^43^ These results demonstrate that rGO incorporation effectively enhances the photoelectrical properties of CZTS, making the CZTS/rGO nanocomposite a promising material for solar-energy conversion applications.

## 4. Conclusion

In summary, we have successfully prepared CZTS/rGO nanocomposites by using a simple, non-toxic, solution-based method. The results of this study show that annealing temperature induced an increase in both crystallinity and dislocation density in the CZTS material. However, the CZTS material annealed at 500 °C, with a crystallite size of 8.15 nm based on the XRD analysis, was used to prepare the CZTS/rGO nanocomposite. The (112), (220), and (200) planes were observed in the XRD pattern of the CZTS/RGO nanocomposite, which is well supported by the SAED pattern and HRTEM fringes. The microstructural study provides evidence for the incorporation of rGO in the CZTS material. The optical analysis revealed that the band gap energy (*E*_g_) of the CZTS/rGO nanocomposite system was 1.47 ± 0.02 eV, which is lower than that of the CZTS nanomaterial (*E*_g_ = 1.49 ± 0.05 eV). The observed improved photocurrent of the CZTS/RGO nanocomposite compared with that of the pristine CZTS revealed an enhanced charge-carrier transport mechanism, improved interfacial charge separation, and suppressed electron–hole recombination. Overall, these results demonstrate that the CZTS/rGO nanocomposite is a promising candidate for use as an active absorber layer in photovoltaic devices, owing to its favorable optical properties and enhanced photoresponse, which may contribute to improved solar-cell performance.

## Conflicts of interest

There are no conflicts to declare.

## Data Availability

The data will be made available upon request.
